# Estimating the burden of SARS-CoV-2 in France

**DOI:** 10.1126/science.abc3517

**Published:** 2020-05-13

**Authors:** Henrik Salje, Cécile Tran Kiem, Noémie Lefrancq, Noémie Courtejoie, Paolo Bosetti, Juliette Paireau, Alessio Andronico, Nathanaël Hozé, Jehanne Richet, Claire-Lise Dubost, Yann Le Strat, Justin Lessler, Daniel Levy-Bruhl, Arnaud Fontanet, Lulla Opatowski, Pierre-Yves Boelle, Simon Cauchemez

**Affiliations:** 1 Mathematical Modelling of Infectious Diseases Unit, Institut Pasteur, UMR2000, CNRS, Paris, France.; 2 Department of Genetics, University of Cambridge, Cambridge, UK.; 3 Department of Epidemiology, Johns Hopkins Bloomberg School of Public Health, Baltimore, MD, USA.; 4 Collège Doctoral, Sorbonne Université, Paris, France.; 5 DREES, Ministère des Solidarités et de la Santé, Paris, France.; 6 Santé Publique France, French National Public Health Agency, Saint-Maurice, France.; 7 Emerging Diseases Epidemiology Unit, Institut Pasteur, Paris, France.; 8 PACRI Unit, Conservatoire National des Arts et Métiers, Paris, France.; 9 Epidemiology and Modelling of Antibiotic Evasion Unit, Institut Pasteur, Paris, France.; 10 Anti-infective Evasion and Pharmacoepidemiology Team, CESP, Université Paris-Saclay, UVSQ, INSERM U1018, Montigny-le-Bretonneux, France.; 11 Institut Pierre Louis d’Epidémiologie et de Santé Publique, Sorbonne Université, INSERM, Paris, France.

## Abstract

France has been heavily affected by the severe acute respiratory syndrome coronavirus 2 (SARS-CoV-2) pandemic and went into lockdown on 17 March 2020. Using models applied to hospital and death data, we estimate the impact of the lockdown and current population immunity. We find that 2.9% of infected individuals are hospitalized and 0.5% of those infected die (95% credible interval: 0.3 to 0.9%), ranging from 0.001% in those under 20 years of age to 8.3% in those 80 years of age or older. Across all ages, men are more likely to be hospitalized, enter intensive care, and die than women. The lockdown reduced the reproductive number from 2.90 to 0.67 (77% reduction). By 11 May 2020, when interventions are scheduled to be eased, we project that 3.5 million people (range: 2.1 million to 6.0 million), or 5.3% of the population (range: 3.3 to 9.3%), will have been infected. Population immunity appears to be insufficient to avoid a second wave if all control measures are released at the end of the lockdown.

## COVID-19 pandemic in France

Coronavirus disease 2019 (COVID-19) exacted a heavy toll in France during March and April 2020. Quarantine measures were effective in reducing transmission by 84%, and some relaxation of social isolation was expected in May. Salje *et al.* fit transmission models for the epidemic in France to hospital admissions. The authors forecast that 2.9 million people will have been infected by 11 May, representing 4.4% of the population—a value inadequate for herd immunity. Daily critical care hospitalizations should reduce from several hundreds to tens of cases, but control will remain a delicate balancing act. Any relaxation of lockdown in France will have to be carefully controlled and monitored to avoid undermining more optimistic forecasts.

*Science*, this issue p. 208

## Teaser

Analysis of COVID-19 epidemiological parameters in France shows clear age and sex patterns that are likely to be common to other countries.

The worldwide pandemic of severe acute respiratory syndrome coronavirus 2 (SARS-CoV-2), the coronavirus that causes coronavirus disease 2019 (COVID-19), has resulted in unprecedented responses, with many affected nations confining residents to their homes. Much like the rest of Europe, France has been hit hard by the pandemic and went into lockdown on 17 March 2020. It was hoped that this lockdown would result in a sharp decline in ongoing spread, as was observed when China locked down after the initial emergence of the virus (*1*, *2*). In light of the expected reduction in cases, the French government has announced it will ease restrictions on 11 May 2020. To exit from the lockdown without escalating infections, we need to understand the underlying level of population immunity and infection, identify those most at risk for severe disease, and determine the impact of current control efforts.

Daily reported numbers of hospitalizations and deaths provide only limited insight into the state of the pandemic. Many people will either develop no symptoms or symptoms so mild that they will not be detected through health care–based surveillance. The concentration of hospitalized cases in older individuals has led to hypotheses that there may be widespread “silent” transmission in younger individuals (*3*). If most of the population were infected, viral transmission would slow, potentially reducing the need for the stringent intervention measures currently employed.

We present a suite of modeling analyses to characterize the dynamics of SARS-CoV-2 transmission in France and the impact of the lockdown on these dynamics. We elucidate the risk of SARS-CoV-2 infection and severe outcomes by age and sex, and we estimate the current proportion of the national and regional populations that have been infected and might be at least temporarily immune (*4*). These models support health care planning of the French government by capturing hospital bed capacity requirements.

**Fig. 1:**
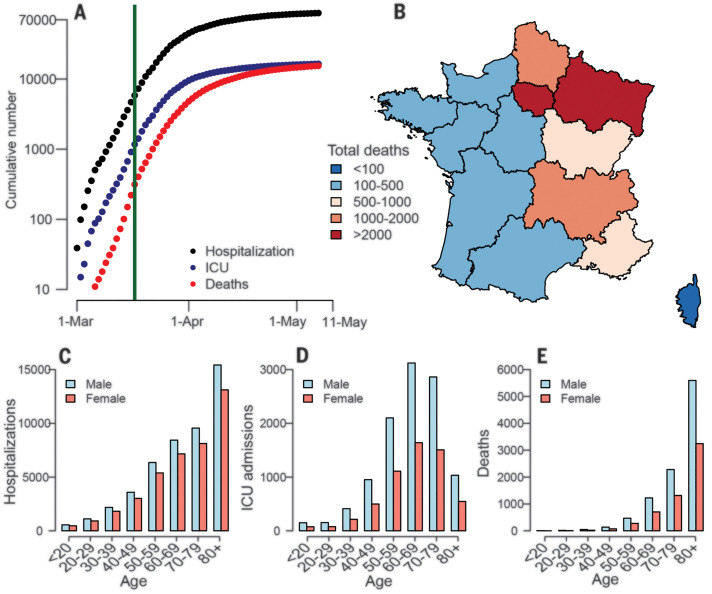
COVID-19 hospitalizations and deaths in France. (**A**) Cumulative number of general ward and ICU hospitalizations, ICU admissions, and deaths from COVID-19 in France. The vertical green line indicates the time when the lockdown was put in place in France. (**B**) Geographical distribution of deaths in France. Number of (**C**) hospitalizations, (**D**) ICU admissions, and (**E**) deaths by age group and sex in France.

As of 7 May 2020, there were 95,210 incident hospitalizations due to SARS-CoV-2 reported in France and 16,386 deaths in hospitals, with the east of the country and the capital, Paris, particularly affected (Fig. 1, A and B). The mean age of hospitalized patients was 68 years and the mean age of the deceased was 79 years, with 50.0% of hospitalizations occurring in individuals over 70 years of age and 81.6% of deaths within that age bracket; 56.2% of hospitalizations and 60.3% of deaths were male (Fig. 1, C to E). To reconstruct the dynamics of all infections, including mild ones, we jointly analyze French hospital data with the results of a detailed outbreak investigation aboard the Diamond Princess cruise ship where all passengers were subsequently tested [719 infections, 14 deaths currently, with one passenger still in the intensive care unit (ICU) after 2 months, who we assume will not survive his infection]. By coupling the passive surveillance data from French hospitals with the active surveillance performed aboard the Diamond Princess, we disentangle the risk of hospitalization for those infected from the underlying probability of infection (*5*, *6*).

**Fig. 2:**
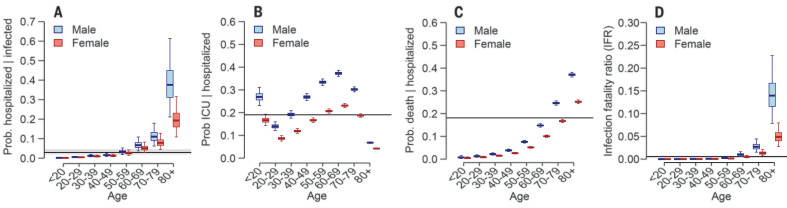
Probabilities of hospitalization, ICU admission, and death. (**A**) Probability of hospitalization among those infected as a function of age and sex. (**B**) Probability of ICU admission among those hospitalized as a function of age and sex. (**C**) Probability of death among those hospitalized as a function of age and sex. (**D**) Probability of death among those infected as a function of age and sex. For each panel, the horizontal black line and gray shaded region represent the overall mean across all ages. The boxplots represent the 2.5, 25, 50, 75, and 97.5 percentiles of the posterior distributions.

We find that 2.9% of infected individuals are hospitalized [95% credible interval (CrI): 1.7 to 4.8%], ranging from 0.1% (95% CrI: 0.1 to 0.2%) in females under 20 years of age to 37.6% (95% CrI: 21.1 to 61.3%) in males 80 years of age or older (Fig. 2A and table S1). On average, 19.0% (95% CrI: 18.7 to 19.4%) of hospitalized patients enter the ICU after a mean delay of 1.5 days (fig. S1). We observe an increasing probability of entering the ICU with age—however, this probability drops for those over 70 years of age (Fig. 2B and table S2). Overall, 18.1% (95% CrI: 17.8 to 18.4%) of hospitalized individuals do not survive (Fig. 2C). The overall probability of death among those infected [the infection fatality ratio (IFR)] is 0.5% (95% CrI: 0.3 to 0.9%), ranging from 0.001% in those under 20 years of age to 8.3% (95% CrI: 4.7 to 13.5%) in those 80 years of age or older (Fig. 2D and table S2). Our estimate of overall IFR is similar to other recent studies that found IFR values between 0.5 and 0.7% for the pandemic in China (*6*–*8*). We find that men have a consistently higher risk than women of hospitalization [relative risk (RR): 1.25; 95% CrI: 1.22 to 1.29], ICU admission once hospitalized (RR: 1.61; 95% CrI: 1.56 to 1.67), and death after hospitalization (RR: 1.47; 95% CrI: 1.42 to 1.53) (fig. S2).

We identify two clear subpopulations among hospitalized cases: individuals that die quickly after hospital admission (15% of fatal cases, with a mean time to death of 0.67 days) and individuals who die after longer time periods (85% of fatal cases, with a mean time to death of 13.2 days) (fig. S3). The proportion of fatal cases who die rapidly remains approximately constant across age groups (fig. S4 and table S3). Potential explanations for different subgroups of fatal cases include heterogeneous patterns of health care seeking, access to care, and underlying comorbidities, such as metabolic disease and other inflammatory conditions. A role for immunopathogenesis has also been proposed (*9*–*12*).

**Fig. 3:**
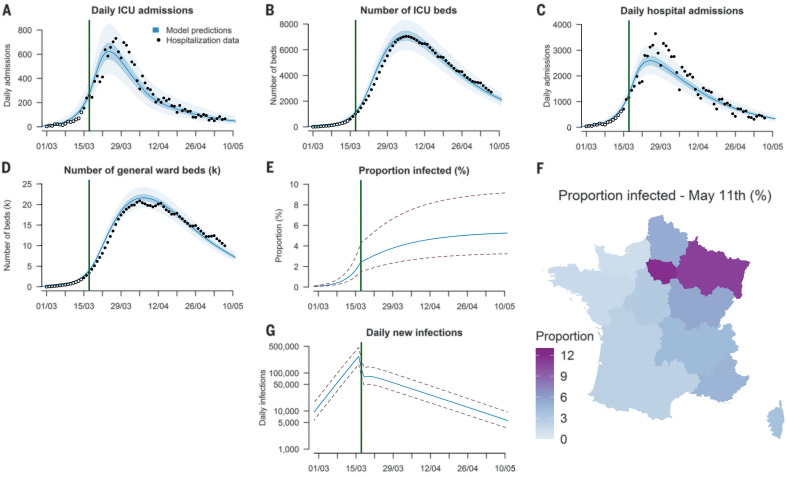
Time course of the SARS-CoV-2 pandemic to 11 May 2020. (**A**) Daily ICU admissions in metropolitan France. (**B**) Number of ICU beds occupied in metropolitan France. (**C**) Daily hospital admissions in metropolitan France. (**D**) Number of general ward beds (in thousands) occupied in metropolitan France. (**E**) Daily new infections in metropolitan France (logarithmic scale). (**F**) Predicted proportion of the population infected by 11 May 2020 for each of the 13 regions in metropolitan France. (**G**) Predicted proportion of the population infected in metropolitan France. The solid circles in (A) to (D) represent hospitalization data used for the calibration, and the open circles represent hospitalization data that were not used for calibration. The dark-blue shaded areas correspond to 50% credible intervals, and the light-blue shaded areas correspond to 95% credible intervals. The dashed lines in (E) and (G) represent the 95% uncertainty range stemming from the uncertainty in the probability of hospitalization after infection.

We next fit national and regional transmission models to ICU admission, hospital admission, and bed occupancy (both ICU and general wards) (Fig. 3, A to D; fig. S5; and tables S4 to S6), allowing for reduced age-specific daily contact patterns following the lockdown and changing patterns of ICU admission over time (fig. S18). We find that the basic reproductive number *R*_0_ before the implementation of the lockdown was 2.90 (95% CrI: 2.81 to 3.01). The lockdown resulted in a 77% (95% CrI: 76 to 78%) reduction in transmission, with the reproduction number *R* dropping to 0.67 (95% CrI: 0.66 to 0.68). We forecast that by 11 May 2020, 3.5 million people (range: 2.1 million to 6.0 million; when accounting for uncertainty in the probability of hospitalization after infection) will have been infected, representing 5.3% (range: 3.3 to 9.3%) of the French population (Fig. 3E). This proportion will be 11.9% (range: 7.6 to 19.4%) in Île-de-France, which includes Paris, and 10.9% (range: 6.9 to 18.1%) in Grand Est, the two most affected regions of the country (Fig. 3F and fig. S5). Assuming a basic reproductive number of *R*_0_ = 2.9, 66% of the population would have to be immune for the pandemic to be controlled by immunity alone. Our results therefore strongly suggest that, without a vaccine, herd immunity on its own will be insufficient to avoid a second wave at the end of the lockdown. Efficient control measures need to be maintained beyond 11 May.

Our model can help inform the ongoing and future response to COVID-19. National ICU daily admissions have gone from 700 at the end of March to 66 on 7 May. Hospital admissions have declined from 3600 to 357 over the same time period, with consistent declines observed throughout France (fig. S5). By 11 May, we project 4700 (range: 2900 to 7900) daily infections across the country, down from between 180,000 and 490,000 immediately before the lockdown. At a regional level, we estimate that 57% of infections will be in Île-de-France and Grand Est combined. We find that the length of time people spend in the ICU appears to differ across the country, which may be due to differences in health care practices (table S5).

**Fig. 4:**
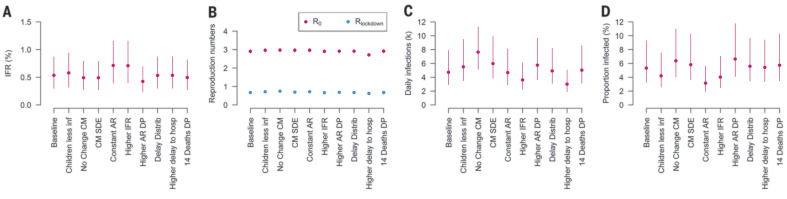
Sensitivity analyses considering different modeling assumptions. (**A**) Infection fatality rate (%). (**B**) Estimated reproduction numbers before (*R*_0_) and during lockdown (*R*_lockdown_). (**C**) Predicted daily new infections on 11 May. (**D**) Predicted proportion of the population infected by 11 May. The different scenarios are as follows: “Children less inf,” individuals under 20 years of age are half as infectious as adults; “No Change CM,” the structure of the contact matrix (CM) is not modified by the lockdown; “CM SDE,” contact matrix after lockdown with very high social distancing of the elderly; “Constant AR,” attack rates are constant across age groups; “Higher IFR,” French people are 25% more likely to die than Diamond Princess passengers; “Higher AR DP,” 25% of the infections were undetected on the Diamond Princess cruise ship; “Delay Distrib,”single hospitalization to death delay distribution; “Higher delay to hosp,” 8 days on average between symptom onset and hospitalization for patients who will require ICU admission and 9 days on average between symptom onset and hospitalization for the patients who will not; “14 Deaths DP,” the final passenger of the Diamond Princess in ICU survives. For estimates of IFR and reproduction numbers before and during lockdown, we report 95% credible intervals. For estimates of daily new infections and proportion of the population infected by 11 May, we report the 95% uncertainty range stemming from the uncertainty in the probability of hospitalization given infection.

Using our modeling framework, we are able to reproduce the observed number of hospitalizations by age and sex in France and the number of observed deaths aboard the Diamond Princess (fig. S6). As a validation, our approach is also able to correctly identify parameters in simulated datasets where the true values are known (fig. S7). As cruise ship passengers may represent a different, healthier population than average French citizens, we run a sensitivity analysis where Diamond Princess passengers are 25% less likely to die than French citizens (Fig. 4 and fig. S8). We also run sensitivity analyses for the following scenarios: longer delays between symptom onset and hospital admission; missed infections aboard the Diamond Princess; a scenario in which the final Diamond Princess patient in the ICU survives; equal attack rates across all ages; reduced infectivity in younger individuals; a contact matrix with unchanged structure before and during the lockdown; and a contact matrix with very high isolation of elderly individuals during the lockdown. These different scenarios result in mean IFRs from 0.4 to 0.7%, the proportion of the population infected by 11 May 2020 ranging from 1.9 to 11.8%, the number of daily infections at this date ranging from 1900 to 11,300, and a range of post-lockdown reproductive numbers of 0.62 to 0.74 (Fig. 4, figs. S8 to S15, and tables S7 to S12).

A seroprevalence of 3% (range: 0 to 3%) has been estimated among blood donors in Hauts-de-France, which is consistent with our model predictions (range: 1 to 3%) for this population if we account for a 10-day delay for seroconversion (*13*, *14*). Future additional serological data will help to further refine estimates of the proportion of the population infected.

Although we focus on deaths occurring in hospitals, there are also nonhospitalized COVID-19 deaths, including >9000 in retirement homes in France (*15*). We explicitly removed the retirement home population from our analyses, as transmission dynamics may be different in these closed populations. This omission means that our estimates of immunity in the general population are unaffected by deaths in retirement homes, however, in the event of large numbers of nonhospitalized deaths in the wider community, we would be underestimating the proportion of the population infected. Analyses of excess death will be important to explore these issues.

This study shows the massive impact that the French lockdown has had on SARS-CoV-2 transmission. Our modeling approach has allowed us to estimate underlying probabilities of infection, hospitalization, and death, which are essential for the interpretation of COVID-19 surveillance data. The forecasts we provide can inform lockdown exit strategies. Our estimates of a low level of immunity against SARS-CoV-2 indicate that efficient control measures that limit transmission risk will have to be maintained beyond 11 May 2020 to avoid a rebound of the pandemic.

## Supplementary Materials

science.sciencemag.org/content/369/6500/208/suppl/DC1 Materials and Methods Supplementary Text Figs. S1 to S18 Tables S1 to S12 References (*17*–*32*)
